# Supplementary material for: Associations of adverse maternal experiences and diabetes on postnatal maternal depression and child social-emotional outcomes in a South African community cohort
Source: PLOS Glob Public Health. 2022 Oct 5;2(10):e0001124. doi: 10.1371/journal.pgph.0001124 (PMC10021654; doi:10.1371/journal.pgph.0001124)
Supplement: Attachment — Submitted filename: Rayport, Response to Editor and Reviewers.docx [file pgph.0001124.s001.docx]

Response to Reviewers

We are thankful to the editor and to the reviewers for their thoughtful comments on our manuscript. We have provided both the track changed version and a clean version of the revised manuscript. As requested, below, we have provided a response to each editor and reviewer comment and identified the page and paragraph numbers where the changes (indicated in blue) have been made to the clean revised manuscript. Additionally, minor edits were made to the manuscript for consistency, clarification and simplification and are noted in the track changed version of the revised manuscript. The changes suggested by the reviewers have improved the overall quality of the manuscript.

**Editor(s)' Comments to the Authors:**

Journal Requirements:

1. In the online submission form, you indicated that your data will be submitted to the Dryad database upon acceptance. Should your submission be accepted, we will require the following information in your Data Availability Statement:

a. The DOI provided by Dryad
b. The citation for your data package in the reference section of your manuscript
c. The citation for your data package in the methods section

If you are unable to adhere to our open data policy, please kindly revise your statement to explain your reasoning and we will seek the editor's input on an exemption. Please be assured that, once you have provided your new statement, the assessment of your exemption will not hold up the peer review process."

**Response:** If the manuscript is accepted for publication, our data will be submitted to the Dryad database to meet the Journal’s criteria.

2. We do not publish any copyright or trademark symbols that usually accompany proprietary names, eg (R), (C), or TM (e.g. next to drug or reagent names). Please remove all instances of trademark/copyright symbols throughout the text, including Questionnaires® on page 7.

**Response:** We removed instances of trademark/copyright symbols.

**Reviewers' comments:**

Reviewer #1: Important elements are missing in the methods section. The authors also need to reorganize some paragraphs (specified in a separate file attached herewith) to improve coherence of ideas. They should also avoid redundancies.

**Response:** Thank you for your feedback – I have responded to your comments from the separate file, pasted below.

Abstract

- It is well constructed, but I suggest checking for minor edits. For instance, the second statement of Conclusion, “Analyses stratified by maternal diabetic status during pregnancy suggest ….”, is an example of unnecessary details for the section repeating what has been mentioned in the Results.

**Response:** Thank you for your suggestion. The statement regarding stratification in the conclusion was simplified (page 2).

1. Introduction

It is uncommon to report evidence gap outright in the second statement of the section (i.e., correlation between metabolic disorders and postnatal maternal depression). It seems the statement appeared prematurely.

The same is true with:

- Part of the first statement of second paragraph for other study variables, i.e., association between poverty and traumatic life events and postnatal maternal depression.
- The first statement of paragraph 4 for the relationship between adverse maternal experiences, diabetes during pregnancy, postnatal maternal depression, and child social-emotional development.

So, I suggest the authors to pool out all the knowledge gap statements (for various variable combination) and synthesize in a concluding paragraph.

**Response:** Thank you for your feedback. We pooled out all the knowledge gap statements and synthesized them in the concluding paragraph (page 4, paragraph 3).

The authors also need to check the section for coherence of information. Reporting reviews from HICs in the last statement of second paragraph after having reported reviews from South Africa in the preceding statements is an example.

**Response:** We checked the introduction for coherence in general and replaced the HIC citation with another example from South Africa (page 4, paragraph 2).

2. Methods

2.1. Population

- Selection of the Bishop Lavis community at their Health Center or the Diabetes Clinic in Tygerberg Academic Hospital is not justified. Preference to an Academic Hospital is not justified as well.

**Response:** We agree that the selection of the health center / clinic was not justified, and we agree with your comment. This study was built on existing collaborations with the university through the Safe Passage Study. This population has been found to have high rates of depression and anxiety (page 5, paragraph 1).

- The use of abbreviations should be checked in the entire manuscript. For instance, GDM is introduced in the very first statement of the Introduction Section to represent gestational diabetes mellitus. However, it is common to see both the abbreviation and its expanded form in subsequent sections, e.g., see the second statement of this sub-section. In another example, Life Events Checklist (LEC) is first introduced in sub-section 2.2. However, it is misused in the fifth statement of sub-section 2.3.

**Response:** The use of abbreviation has been checked throughout the manuscript (page 6).

- Inclusion and exclusion criteria are missing both for mothers and infants and both for cases and comparison groups.

**Response:** We included inclusion and exclusion criteria for both mothers and infants (page 5).

- The authors should also provide a detailed account of how the participants (both mothers and infants and both for cases and comparison groups) were recruited and screened.

**Response:** We included details of how the participants were recruited and screened (page 5).

- It is uncommon to present about ethical considerations along with the description of study population (see the last two statements).

**Response:** The ethics statement was given its own section (page 8).

2.4. Toddler Developmental Assessments

- The authors should check coherence of the information presented under the Methods Section. It doesn’t seem logical to insert the descriptions about Risk Factors in between the descriptions about the two data collection instruments. Thus, this subsection should be moved to precede sub-section 2.3.

**Response:** The risk factors subsection was moved above the self-reported maternal questionnaires subsection (page 6).

2.5. Statistical Analysis

- Given the sample size of 82 mother-infant dyads and 53 women with GDM were included, the authors did not provide any information about data cleaning and management actions in preparation for linear regression models. For example, normality of data distribution, and any measure taken if not normally distributed.

**Response:** All continuous variables (i.e., maternal age, EPDS score, BITSEA score, ASQ:SE:2 score) were normally distributed.

3. Results

3.1. Cohort Characteristics

- Although the authors referred readers to Table 1 for detailed information, the textual information is generally shallow. As an introductory information, it doesn’t cover the characteristics of infants and the comparison groups.

**Response:** More detail was added to the cohort characteristics subsection and now includes characteristics of infants and the comparison groups (page 9).

3.2. Correlates of depression

- Last paragraph needs to be formatted to separate the Figure title from its description.

3.3. Correlates of clinical depression

- Second paragraph needs to be formatted to separate the Figure title from its description.

3.7. Correlates of depression stratified by maternal diabetes status

- Last paragraph needs to be formatted to separate the Figure title from its description.

3.8. Correlates of BITSEA Problem Score stratified by diabetes

- Last paragraph needs to be formatted to separate the Figure title from its description.

3.9. Correlates of BITSEA Competence Score stratified by diabetes

- Last paragraph needs to be formatted to separate the Figure title from its description.

**Response:** Figure titles were separated from the legends (page 14-22).

4. Discussion

4.1. Summary

- Except the first paragraph, all other summaries are repetition of what would be concluded. So, I suggest the authors remove them or synthesize with the conclusion section instead. This will help reduce redundancies with the proceeding detailed discussions.

**Response:** We have synthesized our summaries to help reduce redundancies (page 23).

- There are redundant or irrelevant information for the Section. For example, part of statement 4, “…. also measured by the BITSEA, ….” is a methodological piece of information than a key finding.
- Another similar illustrative example is the first statement of paragraph 3: “To investigate whether diabetes moderates the relationship between the maternal experiences and depression, the cohort was stratified by maternal diabetic status during pregnancy.”

**Response:** Redundant methodological pieces of information were removed from the discussion (page 24).

4.3. Maternal Diabetes as a moderator

- There is repetition of results in paragraph one.

**Response:** We synthesized the discussion to help reduce redundancies (page 25).

Reviewer #2: I want to appreciate the authors for their effort and time in investing more in asses this cohort study in the context of South Africa. The Background, the result, and conclusions of this manuscript are well organized and addressed well but the method section and the discussion need to address and improve. In addition to this editorial errors in the paper, tables and figures also need to address. Here are some comments that need to be addressed.
Background (abstract section): It is better if you say something in the background instead of listing the objective only.

**Response:** Thank you for your suggestion. We added some more background information to the abstract (page 2).

Introduction: In the first paragraph you said that "To the best of our knowledge, there have been no studies ………" Remove this part (you stated on the last paragraph of the introduction).

**Response:** We removed the knowledge gap statement from the first paragraph (page 3).

Methods: This section is good but you failed to address deeply about the study area, sample size, selection criteria, and measuring tool (you addressed but not listed from where did you take (citation)) for the risk factors (Title Number 2.3).

**Response:** We appreciate your comment. We included more detail about the study area, sample size, and selection criteria. The measuring tool for the risk factors (i.e. income, food insecurity), were study-specific maternal questionnaires and there is no citation available (pages 5-7).

In the first section of the Methods Include briefly about the study area for readers. Put the Ethical issue as a subtitle and discuss about the ethical issue in the last section of the methods (including COVID-19). The way that you measured the economic status, physical assault, and food security status is not clear and not cited the reference. Why did you not use wealth index (PCAs))?

**Response:** We included more information on the study area for readers. The ethical statement along with the COVID-19 sentence was given its own subsection and placed at the end of the methods section (pages 5-8).

In the study-specific questionnaires, economic status was measured by asking the mothers’ total monthly household income. There were six possible responses: “less than 500 R,” “500 - 700 R”, “700 – 900 R,” “900-5000 R,” “5000-10,000 R,” and “more than 10,000 R per month.” These six categories were collapsed into four: 0-900 R, 901-5000 R, 5001-10,000 R, to more than 10,000 R per month.

Physical assault was measured with the Life Events Checklist (cited on page 6). In the Life Events Checklist, participants are asked if they have experienced “physical assault (for example, being attacked, hit, slapped, kicked, beaten up).” In response, participants can respond with, “happened to me,” “witnessed it,” “learned about it,” “not sure,” “doesn’t apply.” We collapsed the categories into a binary variable of “happened to me” versus not.

Food insecurity, also from the study-specific questionnaire, was measured by asking the mothers to describe the food in their household in the last 4 months. In response, participants can answer: “Enough of the kind of food we want,” “Enough, but not always the kind of food we want to eat,” “sometimes not enough to eat,” “often not enough to eat.” The last two categories were collapsed due to a small sample.

We did not use a wealth index (PCA) because we wanted to know the individual contribution of each of the socioeconomic indicators, rather than using a summary variable.

Result: The head of the tables needs editing and the percentage (%) written in the table is repeatedly written in the tables (you can write as N (%) at the top of the characteristics (for example Table 1). The way how that you measure the physical assault (as shown in the table) is not clear (how did you measure it)? The way that you measured the food security as shown in the table is also not clear.

**Response:** Percentages were removed throughout the table and included in the headers. More detail was added in regard to how physical assault and food security was measured in the table (pages 9-11).

Discussion: This section is good but you did not discuss the pertinent findings of this study with the other findings (mainly the variables (the summary part)). Include abbreviations, ethics, data availability, funding, statement of conflict of interest, and author's contributions in the last section (above the reference). The figures (Fig 1-5) which are listed below the reference are not visible to read (I am unable to read this part).

**Response:** We reworked the discussion to incorporate other relevant findings with our findings (pages 24-26).

Sections related to abbreviations, ethics, data availability, funding, statement of conflict of interest, and author's contributions were not included in the body of the manuscript per PLOS GPH’s submission guidelines.

We apologize that the figures did not appear clearly. Figures 1-5 appear in low resolution in the submission document. There is a link on the top right of each page to access the original tif, which will reveal the high-resolution figures. We have also included screenshots of the figures on the last pages of the document for ease.

Figure 1: Depression

Figure 2: Child outcomes

Figure 3: Depression

Figure 4: BITSEA

Figure 5: ASQ:SE:2
